# Skeletal Muscle Modulates Huntington’s Disease Pathogenesis in Mice: Role of Physical Exercise

**DOI:** 10.1177/1179069518809059

**Published:** 2018-10-30

**Authors:** Silvia Corrochano, Gonzalo Blanco, Abraham Acevedo-Arozena

**Affiliations:** 1 Mammalian Genetics Unit, MRC Harwell Institute, Oxfordshire, UK; 2 Department of Biology, University of York, York, UK; 3 Unidad de Investigación, Hospital Universitario de Canarias, Fundación Canaria de Investigación Sanitaria e Instituto de Tecnologías Biomédicas, La Laguna, Spain

**Keywords:** Huntington’s disease, skeletal muscle, AMPK, exercise

## Abstract

Huntington’s disease (HD) is a monogenic fatal neurodegenerative disorder. However, there is increasing evidence that HD is a pleiotropic systemic disorder. In particular, skeletal muscle metabolism is greatly affected in HD, which in turn can have a major impact on whole-body metabolism and energetic balance. Throughout an unbiased mutagenesis approach in HD mice, we have found that *Scn4a*, a skeletal muscle–specific sodium channel gene, is a modifier of the disease. Mutations in *Scn4a* enhance HD disease progression and weight loss by accelerating muscle waste and cachexia, increasing skeletal muscle activity and energy demands. At the molecular level, *Scn4a* mutations activate AMP-activated protein kinase (AMPK), leading to a fibre switch towards more oxidative types. These adaptations seen in HD; *Scn4a* double mutant muscles are similar to those observed in healthy individuals after endurance exercise training regimes. This prompted us to assess the effects of an endurance exercise regime in HD mice, independently showing that skeletal muscle adaptations leading to the activation of AMPK are detrimental for HD pathogenesis. Although it is undeniable that physical exercise can lead to many health benefits, our work shows that, at least under certain situations such as in HD, an endurance exercise routine could be a detrimental therapeutic option.

**Comment on:** Corrochano S, Blanco G, Williams D, et al. A genetic modifier suggests that endurance exercise exacerbates Huntington’s disease. *Hum Mol Genet*. 2018;27:1723–1731. doi:10.1093/hmg/ddy077. PubMed PMID:29509900; PubMed Central PMCID:PMC5932560. https://www.ncbi.nlm.nih.gov/pubmed/29509900

It is undeniable that physical exercise can improve health. However, we and others have shown that at least in some situations, such as in disorders that can lead to alterations in skeletal muscle metabolism, including Huntington’s disease (HD), certain types of exercise can accelerate disease onset and progression.

In most neurodegenerative disorders, the brain is not the only affected part of the body, and systemic alterations are evident. This is not surprising, as many of the genes identified as causative of neurodegeneration (eg, the pathological expansions in the first exon of the huntingtin gene responsible for HD) are ubiquitously expressed. In particular, disorders that lead to motor impairments, such as HD and amyotrophic lateral sclerosis (ALS), have prominent systemic alterations. In HD patients and mouse models, the disease seems to increase energy consumption and demands, leading to a hypermetabolic state.^1^ As the energy resources are depleted, muscle wasting and weight loss become indicators of disease progression. Skeletal muscle plays a key role in the regulation of whole-body metabolism, glycaemia, and energy balance. As prominent muscle dysfunction occurs in HD and other neurodegenerative disorders, these systemic alterations are likely to be mediated, at least in part, by dynamic changes in skeletal muscle function through the progression of the neurodegenerative process. These metabolic changes are manifested in skeletal muscle by a switch of muscle fibres towards more oxidative types, typically from type II to type I fibres (glycolytic to oxidative type), accompanied by changes in mitochondrial bioenergetics and biogenesis.

In HD, Parkinson’s disease (PD), ALS, and other neurodegenerative movement disorders, mitochondrial biogenesis and functions are compromised. In particular, lower levels and activity of PGC-1α (peroxisome proliferator–activated receptor-gamma co-activator 1 alpha) have been reported in cellular and animal models and in patients of ALS, PD, and HD.^2–4^It is then not surprising that increasing mitochondrial function is being pursued as a promising therapeutic approach in these neurodegenerative disorders. There is some evidence in the literature suggesting that increasing mitochondrial biogenesis could be beneficial for HD pathogenesis, for example, by overexpressing PGC-1α at the neuronal level in the striatum (and in cellular models).^5,6^ However, these possible beneficial effects may not be generalised to other neurodegenerative conditions. In ALS, overexpression of PGC-1α in skeletal muscle of a mouse model increased mitochondrial biogenesis and muscle function without affecting disease progression, or extending survival,^7^ whereas general overexpression of PGC-1α seems to delay the progression of the disease in a mouse model of ALS.^8^ In Alzheimer’s disease (AD), overexpression of PGC-1α in a mouse model was detrimental for the disease through a mechanism involving impairing autophagy leading to the accumulation of toxic amyloid beta (AB).^9^

In our recent study using HD mouse models,^10^ we have shown that systemic HD pathogenesis can be modulated by local alterations in skeletal muscle that in turn can affect whole-body metabolism. Through an unbiased mutagenesis approach to find new HD modifier genes, we found that mutations in *Scn4a* act as potent enhancers of HD pathogenesis in the mouse. *Scn4a*, the gene encoding the skeletal muscle–specific voltage-gated sodium channel (Nav1.4), is expressed at high levels only in skeletal muscle, whereas the transcript is barely detectable in the brain. This unique pattern of expression allowed us to conclude that the systemic modifier effects were primarily mediated by local changes in HD pathology in skeletal muscle. Overall, the genetic interaction of both mutations (HD and *Scn4a*) led to the exacerbation of the systemic hypermetabolic state of HD mice, making the mice leaner than HD controls from an early age. This was accompanied by a depletion of energy sources from blood, including lower circulating glucose and fatty acid levels in double mutants. Enlarged mitochondria appeared in skeletal muscle of HD mice carrying *Scn4a* mutations, coupled with an almost complete switch towards more oxidative fibre types. Crucially, at least ex vivo, these enlarged mitochondria from the double mutant mice were functional, as when purified they were able to produce more energy than those from their HD littermate controls. All these changes were underlined by the activation of AMP-activated protein kinase (AMPK) in skeletal muscle, coupled with an increase in huntingtin accumulation in skeletal muscle. Altogether, our results suggest that increasing mitochondrial biogenesis and function in skeletal muscle causes further perturbations of the fragile energetic status of HD mice and can lead to deleterious effects on overall HD pathogenesis.

Intriguingly, one of the main adaptations observed in skeletal muscles of individuals that routinely perform endurance exercise training is an increase in mitochondrial biogenesis and bioenergetics remodelling. These adaptations are orchestrated by the activity of major regulators, such as AMPK and PGC-1α, and are coupled with fibre-type switching. Thus, endurance exercise can lead to similar adaptations in healthy individuals than those observed in the HD; *Scn4a* double mutant mice. This observation prompted us to consider that endurance training leading to AMPK activation in skeletal muscle could also be detrimental for HD.

The effects of physical exercise as a possible therapeutic intervention for HD have led to disparaging results. In patients, a recent clinical trial of moderate exercise concluded that it was not detrimental for the disease, although more evidence was needed to assess its possible beneficial effects.^11^ In our study, the effects of endurance exercise were also deleterious for HD pathogenesis. Indeed, different types of exercise routines might exert some beneficial effects on the neurodegenerative process, especially at presymptomatic stages. In particular, aerobic exercise leads to benefits on clinically depressed patients, which is one of the clinical manifestations of HD and other neurodegenerative disorders. Moreover, exercise has been shown to improve cognition through the induction of neurotrophic factors such as brain-derived neurotrophic factor (BDNF) and can induce adult hippocampal neurogenesis.^12^ However, in previous studies using HD mouse models, moderate exercise regimes have not led to any changes in the overall disease onset or progression, despite inducing neurogenesis in the hippocampus.^13^ Thus, it seems that the beneficial effects of exercise are more evident at the cognitive level and increasing neurogenesis, whereas the effects of exercise on overall disease progression remain unclear. Hence, a consistent picture of the effects of physical exercise in different models of neurodegenerative disorders has not yet emerged.

Moreover, mimicking endurance training with pharmacologic compounds in mouse models of HD did not result in locomotor or survival improvement. AICAR is a drug that stimulates the activation of AMPK and is therefore considered to behave as an exercise mimetics drug. AICAR injections in HD mice led to improved cognition and coordination without extending survival in one study, but to increase apoptosis and accelerated death in another.^14^ Moreover, AMPK activation and nuclear translocation in the brain can lead to detrimental effects in HD,^15^ supporting the idea that stimulating mitochondrial biogenesis and the bioenergetics of the cells that are under a critical diseased situation, such as the alterations induced by mutant huntingtin, might not be the most optimal therapeutic option for this disorder. An explanation for the possible detrimental effects of increasing mitochondrial biogenesis in HD could be that although mitochondria seem functional in the HD context, they could also be damaged by the effects of mutant huntingtin, leading to the production of more oxidative stress and damage that could accumulate over time. Indeed, AMPK activation should lead to the activation of autophagy and mitophagy, but this effect is likely compromised in the HD context, as we implied by the accumulation of mutant huntingtin, LC3-II, and the appearance of altered mitochondria in double mutant HD; *Scn4a* skeletal muscles.^10^ Therefore, the possible benefits of incrementing mitochondrial function in HD pathogenesis may be observed only when they are accompanied by a continuous supply of energy in an environment where those mitochondria might not be damaged by the presence of mutant huntingtin.

In summary, increasing energy demands in skeletal muscle through genetic means or by an endurance physical exercise regime, at least in the mouse, are detrimental for HD pathogenesis. This highlights the key role of the periphery in modulating a neurodegenerative disorder like HD. Alterations in skeletal muscle metabolism can have a great impact on systemic metabolism, leading to pleotropic effects observed in the disease. An intriguing possibility is that skeletal muscle dysfunction in HD could lead to changes in the secretion pattern of myokines that mediate the beneficial effects of physical exercise from skeletal muscle to the brain. Clearly, more research is needed to further understand the effects that skeletal muscle dysfunction may have on HD and other neurodegenerative disorders, as well as the effects of physical exercise as a possible therapeutic intervention.
